# Penetrating Neck Trauma: Review of 192 Cases

**DOI:** 10.5812/atr.5308

**Published:** 2012-06-01

**Authors:** Mohsen Mahmoodie, Behnam Sanei, Mohammad Moazeni-Bistgani, Mohammad Namgar

**Affiliations:** 1 Department of Surgery, Faculty of Medicine, Isfahan University of Medical Sciences, Isfahan, IR Iran; 2 Department of Surgery, Faculty of Medicine, Shahrekord University of Medical Sciences, Shahrekord, IR Iran; 3 Faculty of Medicine, Isfahan University of Medical Sciences, Isfahan, IR Iran

**Keywords:** Complications, Mortality, Neck, Penetrating Head Injury, Wound and Injury

## Abstract

**Background:**

The neck region contains a high density of vital organ structures within a relatively small and unprotected anatomic region, making it one of the most vulnerable areas of the body for all types of injuries.

**Objectives:**

In this article, we studied penetrating neck trauma cases in Alzahra Hospital over a 10-year period.

**Patients and Methods:**

In this retrospective, descriptive, analytical study, penetrating neck trauma cases admitted to Alzahra Hospital between April 2000 and April 2010 were analyzed for epidemiology, mechanism of trauma, zone of trauma, therapeutic method, injuries to other organs, complications, and mortality.

**Results:**

Among 192 penetrating neck injuries, the mean age at the time of injury was 25.08 ± 15.02 years. Of these cases, 96.4% occurred in men. The most common mechanisms of trauma was stab wounds (85.93%). In 56.3% of penetrating neck injuries, zone 2 was involved. Neck exploration was positive in 84.4% of cases, and 52.1% of patients underwent surgery. Vascular exploration was the most common cause of surgery (67.2% of patients). The most common surgical intervention was vein ligation (50.8% of cases). In 11.98% of cases, another organ injury occurred simultaneously, and chest injury was the most common coexisting problem (65.2%). Complications were reported in 9.3% of patients, and the need for intubation was the most common complication (5.2% of patients). Mortality rate was 1.5%.

**Conclusions:**

According to the findings of this study, the most common cause of penetrating neck injuries was stab wounds, and the majority of patients were young men, therefore, preventive measures should be implemented. Because of fatal complications associated with neck injuries, we recommend early neck exploration in unstable cases or when injuries are deeper than the platysma.

## 1. Background

The neck region contains a high density of vital organ structures in a relatively small and unprotected anatomic region, making it one of the most vulnerable areas of the body for all types of injuries (1). Injuries to the neck can be secondary to both blunt and penetrating trauma (2). Penetrating neck wounds are present in approximately 10% of all trauma patients (3). While penetrating neck wounds are most commonly associated with violent acts, they are also encountered in road traffic collisions and other accidents. The mechanism of penetration is important in determining the extent of damage and treatment options. Ballistic missile trauma can cause extensive damage that is highly correlated with the velocity of the projectile. Stab wounds are relatively low velocity, but can still lead to serious injury. It is useful to divide the neck horizontally into 3 zones. Zone I extends between the clavicles and the cricoid cartilage; injuries to this zone carry the highest mortality because of vascular injury and high-risk surgical explorations (4). Zone II is superior to Zone I and extends as far as the angle of the mandible. Zone III is the area between the angle of the mandible and the base of the skull. Zone II injuries are the most common, followed by Zone I, and then Zone III (5). Inevitably, vascular injury is the most frequent complication of penetrating neck trauma, occurring in one-quarter of all cases, and carrying a mortality of nearly 50%. Trauma to the trachea occurs in one-tenth of cases, and mortality in these cases approaches 20% (6). Other structures at risk of damage include the cranial nerves and esophagus, in which damage can cause leakage of digestive enzymes and bacteria into surrounding tissues. Extensive penetration may result in oropharyngeal trauma. Currently, it is believed that penetrating neck injuries carry a 3–6% mortality rate (7). Lydiatt revealed that, in the United States, most penetrating neck traumas in the adult population are secondary to assaults. Some cases of penetrating neck trauma in adults and most cases in children are caused by accidents, such as falls on sharp objects and motor vehicle accidents. Accidental penetrating injuries are uncommon and their actual frequency rates are not reported. Internationally, the rate of penetrating neck trauma is usually related to the violent crime rate and military conflicts in a particular country. The most common causes of penetrating neck trauma are missile injuries from firearms and stab injuries. Accidental penetrating injuries are most often due to falls on sharp objects, such as sticks or glass. Motor vehicle accidents are another cause of accidental penetrating injuries. The current mortality rate for penetrating neck injury is 3–6%, with 50% of deaths caused by hemorrhage from vascular injuries. Vascular injuries cause complications in 40% of cases of penetrating neck injuries, and 10% of patients have an injury to the carotid artery. Aerodigestive tract injuries occur in 23–30% of patients with penetrating neck wounds, and esophageal injuries are associated with mortality rates of 11–17% (8). In a study by Roden and Pomerantz at Northwestern University Medical School, out of a total of 30 patients, 12 underwent immediate operative exploration based upon clinical indications present at admission. Seventeen patients underwent further diagnostic testing, including angiography in 17 and contrast esophagography in 8. Endoscopy was used infrequently. The mortality rate was 13.3%, and there were 2 negative cervical explorations and no missed injuries. The results support the application of a selective approach to the operative management of penetrating injuries to the neck (9). According to another study by Narrod and Moore, during the first 6 h after injury, all injuries were explored. Of 75 patients explored, only 33 (44%) had significant injuries. In reports from the last 4 years, patients were managed selectively. Patients with bleeding, crepitation, dysphagia, or compromised airways, or for those in whom full clinical evaluation was not possible underwent prompt formal operative exploration. All other patients were observed. Of the 48 patients who underwent explorations, 41 (85%) had significant injuries. Thirty-six patients were observed with no adverse sequelae. Ancillary diagnostic testing was only routinely conducted in Zone I injuries. Over the last 10 years, we evolved from mandatory exploration to selective exploration of anterior penetrating neck injuries. Our experience confirms the safety and cost-effectiveness of a selective exploration policy. Furthermore, observation does not mandate extensive ancillary diagnostic testing f Zone II and Zone III injuries (10). According to a study of Bryan et al., 121 consecutive patients were identified from the Legacy Emanuel Trauma Registry as having sustained penetrating neck injuries from 2000 to 2005, 55 of which had only superficial injuries that did not penetrate the platysma. The primary study group consisted of 65 patients who sustained more significant injuries that violated the platysma, including deep, complex, and/or avulsion wounds, vascular injuries, and injuries to the aerodigestive tract, musculoskeletal system, cranial nerves, or thyroid gland. The overall mortality rate for the 65 patients with injuries penetrating the platysma was 3.0% (n = 2). Complications occurred in 7 of the surviving 63 patients (10.7%) (11).

## 2. Objectives

This retrospective study of penetrating neck trauma may assist physicians in making decisions for prophylactic and better management of these patients. Therefore, we studied penetrating neck trauma cases at Alzahra Hospital that occurred during the 10 years from April 2000 to April 2010.

## 3. Patients and Methods

**Figure 1.**
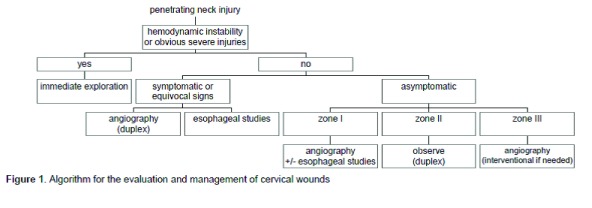
Algorithm for the evaluation and management of cervical wounds.

In this retrospective, descriptive, analytical study, files from all patients who were referred to the hospital because of penetrating neck traumas from April 2000 to April 2010 were reviewed. During these years, our treatment protocol included selective management based on neck zones and angiography, barium swallow, and rigid esophagoscopy analysis (Figure 1). 

Data files were reviewed for age, sex, zone of the trauma, traumatized viscera, mechanism of trauma, complications, type of management, negative neck exploration, and associated trauma, and recorded in the questionnaire. Incomplete files were completed by telephone. Data were analyzed using SPSS version 16 with descriptive tests. 

## 4. Results

Among 192 penetrating neck injuries, the mean age of patients was 25.1 ± 15.0 years (Table 1). Of these patients, 96.4% were men. Mechanisms of trauma included stab wounds (85.93%), gunshot wounds (7.82%), and other injuries (6.25%). Negative neck exploration occurred in 15.6% of cases (Table 1). 

**Table 1.** Patient Data^a^

	No. (%)
Sex	
Male	185 (96.4)
Female	7 (3.6)
Mechanisms	
Stab wound	16 (85.93)
Gunshot	15 (7.82)
Others	12 (6.25)
Trauma zone	
1	51 (26.6)
2	108 (56.3)
3	26 (13.5)
1, 2	2 (1)
2, 3	5 (2.6)
Damaged organ	
Vein	31 (31)
Artery	10 (10)
Esophagus	0 (0)
Pharynx	5 (5)
Laryngotracheal	15 (15)
Associated trauma	
FX limbs^b^	5 (21.7)
Chest	15 (65.2)
Abdomen	3 (13.1)
Pelvic	0 (0)
Negative neck exploration	
Yes	30 (15.6)
No	162 (84.4)
Type of management	
Surgery	100 (52.1)
Conservative	92 (47.9)
Complications	
Intubation	10 (55.6)
Tracheostomy	3 (16.6)
CVA^b^	0 (0)
DVT^b^	5 (27.8)
Infection	0 (0)
Death	3 (1.5)

^a^The age of the study group varied from 5 to 60 years old; Mean ± SD: 25.08 ± 15.02

^b^Abbreviations: CVA, Cerebral vascular artery; DVT, Deep vein thrombosis; FX, Fracture

Penetrating neck injuries occurred most frequently in zone 2 (56.3%), followed by zone 1 (26.6%) and zone 3 (13.5%). The rest of the injuries occurred in multiple zones. Injuries to other organs were present in 11.98% of cases, and chest injury was the most common coexisting problem (65.2%). Furthermore, 52.1% of patients underwent surgery, and conservative management was used in 47.9% of cases. The results of neck exploration were positive in 84.4% of cases. Vascular exploration was the most common cause of surgery (67.2%). Common surgical interventions were vein ligation (50.8%), arterial repair (16.4%), laryngotracheal repair (24.6%), and pharyngeal repair (8.2%). The complication rate was 9.3%. Requirement of intubation was the most common complication (5.2% of patients). Mortality occurred in 1.5% of patients (Table 1). 

## 5. Discussion

The neck is one of the most important topographic areas due to the high density of vital organ structures. Penetrating trauma to the neck causes injury to these structures, resulting in mortality and morbidity. Retrospective studies of penetrating neck trauma help physicians make decisions for the prevention and better management of these patients. In our study, only 3.6% of patients were female, and the mean age of the patients was 25.08 ± 15.02 years. However, in a study by Bryan et al. in 2007 (11), the incidence of penetrating neck trauma in women was 3 times higher than in men, and the mean age of the patients was 33.8 year . These age and gender differences may have been caused by differences in culture between the 2 studies. The young population in our country is the predominant population, and this population is highly active; therefore, penetrating neck trauma is common in this population.

According to findings of our study, most penetrating neck trauma was due to stab injuries (85.93%), while the study by Lydiatt (8), conducted in the United States, found that most penetrating neck trauma in the adult population was secondary to the personal assaults, from firearm and stab injuries. This difference may be due to the laws prohibiting the keeping and carrying of firearms in our country.

In our study, the most common zone of neck injury was zone ІІ (56.3%). This was in accordance with a study by Sriussadaporn et al. in 2002 (12), demonstrating that the most common zone in penetrating neck trauma was zone ІІ (64% of cases). This similarity may be due to the susceptibility of this zone for neck trauma.

In our study, the most commonly injured structures in the neck were the vessels (67.2%), followed by the laryngotracheal region (24.9%) and pharynx (8.2%). However, in a prospective study of 223 patients with penetrating neck injuries in Los Angeles, conducted by Demetriades et al. (13), the most commonly injured structures in the neck were the vessels, followed by the spinal cord, aerodigestive tracts, and nerves. This difference is likely a result of the mechanism of trauma. In this study, the most common mechanism was gunshot, while in our study, the most common mechanism was injury from stab wounds. Because of the proximity to the neck and chest, chest trauma was the most common associated trauma in our study (65.2% of cases); nevertheless, any organ may be injured, since the mechanism of trauma can vary. In our study, 52.1% of patients required neck exploration. However, according to studies by Roden et al. (9), and Gracias (14), 40% and 50% of penetrating neck traumas, respectively, required neck exploration. This difference may have resulted from differences in the severity and mechanisms of trauma. The management protocol requiring mandatory exploration for penetrating neck injuries that violate the platysma emerged from the military during wartimes (15, 16). This policy was based on the favorable results obtained with this approach and the complex anatomy of the neck. High rates of negative neck exploration, up to 75% (17), and the relatively high number of serious injuries overlooked during operations resulted in the critical reappraisal of recommended policies requiring mandatory exploration (16, 18). Therefore, surgeons concluded that using a selective management algorithm avoided unnecessary exploration, regardless of the location of the wound, and some patients with penetrating neck injuries can be safely managed without surgery. Our treatment protocol was based on a selective management protocol according to neck zones, barium swallow test, rigid esophagoscopy (19) and an algorithm of evaluation and management of cervical wounds; however, because of angiography was impossible in our hospital, vascular injury was suggested by history and physical examination. In spite of the fact that angiography was impossible in our hospital, in our study, negative neck exploration was 15.6%. In a series of 4 studies led by Moore in Denver, Colorado, the evolving treatment of penetrating neck wounds can be followed. Overall, 105 patients (34%) underwent surgery early, with a relatively low rate of nontherapeutic explorations (16%). The authors reported a very satisfactory outcome based on an algorithm of evaluation and management of cervical wounds. This result was the same as our study. We concluded that physical examination reliably predicted major vascular trauma similar to 2 additional studies by Demetriades and colleagues (13, 21) that reported the results of a prospective study during a 2-year period in Los Angeles, California. They evaluated the sensitivity of physical examination for identifying patients with penetrating injuries of the neck in patients who required immediate exploration compared to those who needed diagnostic studies (namely arteriography). They also addressed the role of color flow Doppler imaging as an alternative to angiography. During a 2-year study, 223 patients entered the study, and in a group of 34 patients with equivocal clinical signs, only 1 required surgical treatment. The authors concluded that physical examination reliably predicted major vascular trauma.

In our study, complications were reported in 9.3% of cases, and the need for intubation was the most common complication (5.2%), following by deep vein thrombosis (DVT) and the need for tracheostomy. The mortality rate was 1.5% due to associated trauma, but not due to penetrating neck injuries. In contrast, in the study by Bryan and coauthors (11), complications occurred in 7 of the surviving 63 patients (10.7%), including 2 patients with zone 3 internal carotid artery injuries who developed hemispheric ischemic infarcts and hemiplegia and other complications such as infection (n = 2), deep venous thrombosis, aspiration pneumonia, and hematoma (n = 1 for each). All surviving patients, except the 2 stroke patients, eventually healed uneventfully without significant functional deficits. The reason that no patients died in this study may have been due to differences in the mechanisms of trauma.
